# Establishing the Phenolic Composition of *Olea europaea* L. Leaves from Cultivars Grown in Morocco as a Crucial Step Towards Their Subsequent Exploitation

**DOI:** 10.3390/molecules23102524

**Published:** 2018-10-02

**Authors:** Lucía Olmo-García, Aadil Bajoub, Sara Benlamaalam, Elena Hurtado-Fernández, María Gracia Bagur-González, Mohammed Chigr, Mohamed Mbarki, Alberto Fernández-Gutiérrez, Alegría Carrasco-Pancorbo

**Affiliations:** 1 Department of Analytical Chemistry, Faculty of Science, University of Granada, Ave. Fuentenueva s/n, 18071 Granada, Spain; luciaolmo@ugr.es (L.O.-G.); elenahf@ugr.es (E.H.-F.); mgbagur@ugr.es (M.G.B.-G.); albertof@ugr.es (A.F.-G.); 2 Department of Basic Sciences, National School of Agriculture, km 10, Haj Kaddour Road, B.P. S/40, 50001 Meknès, Morocco; aliam80@hotmail.com; 3 Laboratory of Chemical Processes and Applied Materials, Faculty of Science and Technology, University of Sultan Moulay Slimane, BP 523, 23000 Béni Mellal, Morocco; s.benlamaalam@gmail.com (S.B.); chigrm@gmail.com (M.C.); mbarki63@yahoo.fr (M.M.)

**Keywords:** olive leaves, Moroccan region, phenolic compounds, liquid chromatography-mass spectrometry, chemometrics, metabolic profiling

## Abstract

In Morocco, the recovery of olive agro-industrial by-products as potential sources of high-added value substances has been underestimated so far. A comprehensive quantitative characterization of olive leaves’ bioactive compounds is crucial for any attempt to change this situation and to implement the valorization concept in emerging countries. Thus, the phenolic fraction of olive leaves of 11 varieties (‘Arbequina’, ‘Hojiblanca’, ‘Frantoio’, ‘Koroneiki’, ‘Lechín’, ‘Lucque’, ‘Manzanilla’, ‘Picholine de Languedoc’, ‘Picholine Marocaine’, ‘Picual’ and ‘Verdal’), cultivated in the Moroccan Meknès region, was investigated. Thirty eight phenolic or related compounds (including 16 secoiridoids, nine flavonoids in their aglycone form, seven flavonoids in glycosylated form, four simple phenols, one phenolic acid and one lignan) were determined in a total of 55 samples by using ultrasonic-assisted extraction and liquid chromatography coupled to electrospray ionization-ion trap mass spectrometry (LC-ESI-IT MS). Very remarkable quantitative differences were observed among the profiles of the studied cultivars. ‘Picholine Marocaine’ variety exhibited the highest total phenolic content (around 44 g/kg dry weight (DW)), and logically showed the highest concentration in terms of various individual compounds. In addition, chemometrics (principal components analysis (PCA) and stepwise-linear discriminant analysis (s-LDA)) were applied to the quantitative phenolic compound data, allowing good discrimination of the selected samples according to their varietal origin.

## 1. Introduction

Global production of virgin olive oil has steadily increased over the past decades, reaching 3.1 million tons during the 2017/2018 crop season [1,2], which makes olive tree the sixth most relevant oil crop in the world [3]. Furthermore, its undeniable economic importance has induced the expansion of the virgin olive oil agro-industry, but at the same time, has led to the generation (often in geographically concentrated locations) of huge amounts of wastes, so-called olive by-products. Despite the technological efforts, the generation of these residues is ineludible. The olive oil agro-industry produces large amounts of both solid waste (known as olive pomace or olive cake) and high volumes of effluents (known as olive mill wastewater) per year; the amount depends on the olive oil extraction system used [4]. In addition, as a result of olive tree pruning and the washing of harvested olive fruits, considerable amounts of olive leaves (approximately 25 kg per pruned tree and 5% of the total weight of the harvested olive fruits) are accumulated too [5].

Consumer awareness of sustainability and new strict environmental regulations in various Mediterranean countries are the most important drivers in both the development of strategies for an adequate management of olive by-products and the progress regarding recycling and valorization [6,7]. One of these trends is the recovery of functional components or molecules with interesting (bio)activity (health-promoting, therapeutic or cosmetic properties) to be further re-utilized in areas such as food, pharmaceutical and cosmetic industries [8,9,10].

Phenolic compounds are among those bioactive substances occurring at high concentrations in olive by-products. Olive leaves in particular represent an important resource of these components whose bioactivity, anti-oxidant, antimicrobial and anti-inflammatory properties have been extensively demonstrated [11,12]. Several conventional (solvent-based) and more modern assisted extraction techniques (ultrasound, microwave, sub- and supercritical fluid extraction, pressurized liquid extraction, pulsed electric field and high voltage electrical discharge, among others) have been tested for their recovery [13,14,15,16,17,18]. As stated before, the obtained extracts might have many applications in different fields, including, for instance, food additives and preservatives [19,20,21], cosmetics [22], as well as nutraceuticals and pharmaceuticals [23]. As a consequence, over the last years, characterizing olive leaf phenolic profiles has become a challenging and important analytical task in order to provide comprehensive qualitative and quantitative information regarding the occurrence of these compounds. It is quite evident that their reliable analytical determination is an absolutely pivotal and necessary step preceding (and widely conditioning) the potential subsequent recovery. In this regard, very interesting reports dealing with the identification and quantification of phenolic compounds from olive leaves have been published, including the use of gas chromatography (GC), nuclear magnetic resonance spectroscopy (NMR), high performance liquid chromatography (HPLC) coupled to diode array detection (DAD) and/or mass spectrometry (MS), etc.; they have been recently reviewed [24].

The present work was conceived as a first step to develop a thorough recovery approach of phenolic compounds from olive leaves in Morocco, which ranks sixth in the global production of virgin olive oil. Data from 2015 indicate that the Moroccan olive growing area was approximately 998,000 hectares, yielding 1.15 million tons of olive fruits and 120,000 tons of virgin olive oil [25]. Thus, the olive oil agro-industry certainly stands out as one of the driving sectors of the economy of this country. The recovery of bioactive compounds from olive oil by-products might bring additional benefits to the sector, increasing the profitability and adding value to the supply chain. However, there is a gap regarding olive by-products composition since, to the best of our knowledge, the phenolic profile of leaves from olive trees planted in Morocco has not been studied so far. Therefore, one of the main practical objectives of this study was to deeply investigate the phenolic composition of olive leaves obtained from both autochthonous and recently introduced olive cultivars in this country. To better assess the potential of these compounds as varietal markers, the inter-variety phenolic composition variability was checked. Moreover, chemometric tools were employed to discriminate among the studied cultivars based on the phenolic composition of their leaves. 

## 2. Results and Discussion

### 2.1. Profiling and Qualitative Characterization of the Phenolic Fraction of Olive Leaves from the Selected Eleven Cultivars 

**Figure 1:**
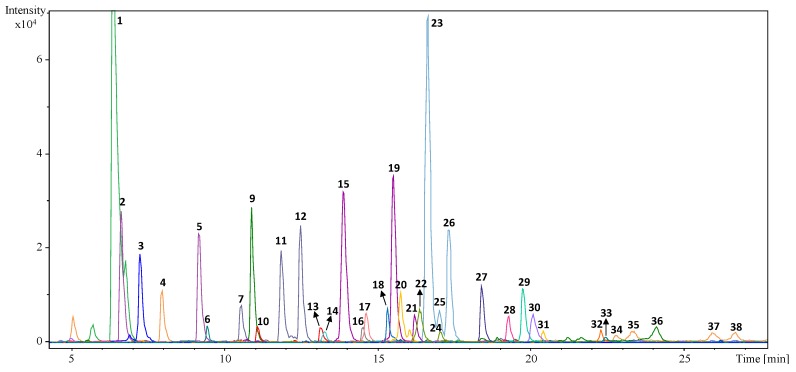
Extracted ion chromatograms (EICs) of the main phenolic compounds identified in a ‘Picholine Marocaine’ olive leaves sample. Numbers correspond with those included in Table 1.

The first stage of this work was designed to carry out a comprehensive characterization of the phenolic profiles of the leaves from different olive varieties, trying to identify as many compounds as possible. Tentative identifications were achieved by considering the information provided by the two detectors (DAD (UV*-vis* spectra) and MS (*m*/*z* spectral data)), the data achieved for the commercial standards (when available), as well as by comparing the information regarding retention time and elution order with the previously published reports [26,27,28,29,30]. Accurate mass data obtained in full-scan mode in a Q-TOF MS was processed with the SmartFormula™ Editor tool included in DataAnalysis 4.0 (Bruker Daltonik, Bremen, Germany), which provides a list of possible elemental formulas. Table 1 lists (according to their elution order) the 38 phenolic compounds tentatively identified in the studied leaves samples and presents the calculated molecular formula for each compound, together with the error (difference between experimental and theoretical *m/z* of the detected [M − H]^−^ ion) and mSigma™ (value showing the concordance with the theoretical isotopic pattern of the compound). Figure 1 shows the Extracted Ion Chromatograms (EICs) of the main identified phenolic compounds found in a sample of ‘Picholine Marocaine’ leaves. 

In general, the phenolic composition of all the investigated samples was dominated by the presence of a high number of different secoiridoids (16 compounds in total) including (in order of elution): Secologanoside isomers 1 (peak **2**) and 2 (peak **5**), elenolic acid glucoside isomers 1, 2 and 3 (peaks **7**, **11** and **12** respectively), oleuropein aglycon isomers 1 and 2 (peaks **9** and **36**, respectively), hydroxyoleuropein (peak **14**), oleuropein diglucoside (peak **17**), 2″-methoxyoleouropein isomers 1 and 2 (peaks **22** and 24 respectively), oleuropein isomers 1 (peak 23), 2 (peak 25) and 3 (peak 26), ligstroside (peak **27**), and ligstroside aglycon (peak **28**) *(readers should note that secologanoside and elenolic acid (and their derivatives) are not strictly phenolic compounds; however, they are usually included under the term “phenolic substances” and we will use this terminology in the current contribution).* Furthermore, the chromatographic profile of the studied samples showed other 16 peaks corresponding to flavonoids (in aglycone or in their glycosylated form). As far as flavonoids in aglycone form are concerned, the group was composed by (in elution order): rutin (peak **13**), luteolin (peak **29**), quercetin (peak **30**), apigenin (peak **32**), naringenin (peak **33**), diosmetin (peak **34**), and three isomers of an unknown compound with calculated molecular formula C_15_H_8_O_7_ (peaks **35**, **37** and 38). In the current report we have decided to include them in this category and quantify them in terms of luteolin (because of their similarity regarding polarity and molecular weight). We logically wanted to compare the concentration levels found in the different cultivars, rather than achieving very accurate quantitative results in absolute terms. Within the group of flavonoids in glycosylated form, we found the following ones: luteolin diglucoside (peak 10), luteolin-7-glucoside (peak 15) and other two luteolin-glucoside isomers (peaks **19** and **21**), apigenin rutinoside (peak **16**), apigenin-7-glucoside (peak **18**), and chrysoeriol-7-glucoside (peak **20**). 

Lastly, it was also possible to find four simple phenols (hydroxytyrosol glucoside (peak **1**), hydroxytyrosol (peak **3**), tyrosol glucoside (peak **4**), and tyrosol (peak **6**)), one phenolic acid (vanillic acid (peak **8**)) and one lignan (pinoresinol (peak **31**)). It should be emphasized that almost all the phenolic compounds identified in the selected samples had been previously reported in very comprehensive papers about the characterization of olive leave extracts [26,27,28,29,30]. However, two aspects distinguish this work from others: the number of compounds determined is greater in comparison, and it represents the first report including the comprehensive profiling of olive leaves from the varieties ‘Lechín’, ‘Lucque’, ‘Picholine de Languedoc’, ‘Picholine Marocaine’ and ‘Verdal’.

### 2.2. Phenolic Contents in Different Olive Leaves Cultivars

Prior to quantifying the identified phenolic compounds, the analytical method was properly validated in terms of linearity, precision (intra- and interday repeatability), limit of detection (LOD) and limit of quantification (LOQ). Thus, as reported in Section 3.2.1, dilutions of the standard solution mixture were prepared and injected into the LC-IT MS system (which was the instrument used for quantifying). Method linearity was evaluated by plotting the peak areas versus the corresponding concentrations (mg/L) of each standard analyte using the least squares method. Calibration curves were built using the values from three replicates of each concentration level analyzed within the same day (*n* = 3). LODs and LOQs of the individual compounds in the standard solutions were calculated as the lowest concentration at which a signal-to-noise (S/N) ratio was greater than 3 and 10, respectively. Intra- and interday repeatability were also estimated; to do it so, we calculated the relative standard deviation (RSD (%)) of peak area for 4 injections of 4 different extracts of the quality control (QC) sample carried out within the same sequence (intraday) or over 4 days (interday). Obtained results for the evaluated analytical parameters are summarized in Table S1 (Supplementary materials).

As shown in the table, linearity of the method was satisfactory over the assayed range with correlation coefficient (r^2^) higher than 0.9918 in all cases. The LODs ranged from 3 to 97 μg/L and the LOQs ranged from 11 to 325 μg/L, for apigenin and rutin, apiece. The method led to excellent precision values (RSD (%)) always lower than 9.4% (values ranged from 1.8% to 7.5% for the *intra-*day repeatability and from 2.1% to 9.4% for the *inter-*day repeatability). Consequently, the proposed analytical method could be successfully applied for the determination of 38 phenolic compounds in the selected 55 olive leaves samples.

Quantification in MS was done using external calibration curves of the corresponding pure standard analytes for: Oleuropein, apigenin, apigenin-7-glucoside, hydroxytyrosol, luteolin, luteolin-7-glucoside, pinoresinol, rutin, tyrosol and vanillic acid, whereas for those identified compounds for which reference pure standards were not available, a calibration curve from structurally related substances was used. Thus, tyrosol glucoside, elenolic acid glucoside isomers (1, 2 and 3), secologanoside isomers (1 and 2) and ligstroside aglycon were quantified using tyrosol calibration curve; hydroxytyrosol glucoside and oleuropein aglycon isomers (1 and 2) were quantified in terms of hydroxytyrosol; apigenin rutinoside and luteolin diglucoside in terms of rutin; chrysoeriol-7-glucoside and luteolin-glucoside isomers (1 and 2) by using luteolin-7-glucoside calibration curve; to quantify oleuropein diglucoside, 2″-methoxyoleoropein isomers (1 and 2), hydroxyoleuropein and ligstroside, the standard of oleuropein was employed; naringenin was determined in terms of apigenin; and finally, quercetin, diosmetin, and the unknown isomers of C_15_H_8_O_7_ were quantified by using luteolin as reference standard. It is important to bear in mind that the response of the standards can differ from the response of the analytes present in the olive leave extract samples, and consequently, the quantification of these compounds (both in terms of total amount and individual contents) is only an estimation of their occurrence in the analyzed samples.

**Figure 2:**
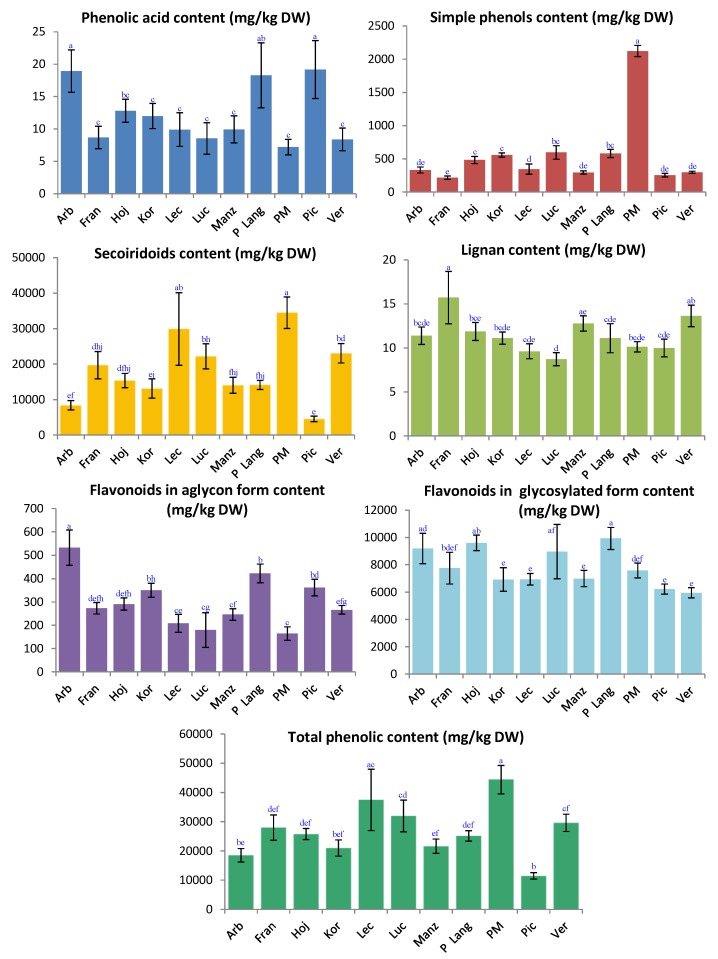
Total phenolic content and content in terms of the different chemical classes (content of secoiridoids, flavonoids in aglycon form, flavonoids in glycosylated form, simple phenols, one phenolic acid and one lignan) of the studied olive leaves samples, expressed in mg/kg DW. Different letters above the bars indicate significant differences at *p* < 0.05, Turkey’s test (comparison among the 11 cultivars investigated in this study). Abbreviations meaning (in alphabetical order): Arb: ‘Arbequina’; Fran: ‘Frantoio’; Hoj: ‘Hojiblanca’; Kor: ‘Koroneiki’; Lech: ‘Lechín’; Luc: ‘Lucque’; Manz: ‘Manzanilla’; P Lang: ‘Picholine de Languedoc’; PM: ‘Picholine Marocaine’; Pic: ‘Picual’; and Verd: ‘Verdal’.

The total phenolic compounds content (sum of the content of individual phenolic compounds determined) and the total phenolic content per chemical class (sum of the content of individual phenolic compounds belonging to the same chemical family) of the olive leaves from the different studied cultivars are given in Figure 2. Results are expressed as mean ± standard deviation. As can be seen, on average terms, total phenolic content ranged from around 11 g/kg DW to 44 g/kg DW; ‘Picual’ was the poorest variety of the studied selection and ‘Picholine Marocaine’ was the richest one. Secoiridoids were by far the most abundant group of phenols in all the analyzed samples regardless of the variety, excepting ‘Arbequina’ and ‘Picual’ samples for which flavonoids (in glycosylated form) were predominant. 

Among the studied cultivars, the highest secoiridoids content (34 g/kg DW) was found in ‘Picholine Marocaine’ leaves extracts, whilst ‘Picual’ samples presented the lowest concentration level (5 g/kg DW). The highest level of total flavonoids in glycosylated form was observed in ‘Picholine de Languedoc’ samples (10 g/kg DW) and the lowest one (6 g/kg DW) in ‘Verdal’ leaves; however, regarding this group of analytes, the differences found among the cultivars were not as noticeable as for others. As far as the other sub-category of flavonoids is concerned, it is possible to highlight that flavonoids in aglycon form were found within the range 165–532 mg/kg DW, defined by ‘Picholine Marocaine’ and ‘Arbequina’, respectively. The content in terms of simple phenols and, in particular, the amounts of vanillic acid and pinoresinol were negligible—in all the cultivars—when compared with secoiridoids levels. In this regard, the concentrations of simple phenols ranged between 218 mg/kg DW and 2124 mg/kg DW, for ‘Frantoio’ and ‘Picholine Marocaine’ leaves extracts, respectively. The content of the quantified lignan was found between 8.7 mg/kg DW (Lucque) and 16 mg/kg DW (‘Frantoio’). Finally, the amount of the phenolic acid fluctuated from 7 mg/kg DW to 19 mg/kg DW; ‘Picholine Marocaine’ and ‘Picual’ exhibited the extreme concentration levels. 

After getting the quantitative results, the existence of significant variations (both regarding total phenolic content and chemical class content) was investigated. One-way ANOVA revealed statistically significant differences among the concentration of phenolic compounds in leaves from different cultivars. Our results support those found in literature with regard to the intervariety variability of the total phenolic content in olive leaves [26,27,30,31]. In general, our quantitative data are also similar to those included in previous reports, even though the comparison in this regard is not very straightforward; it is necessary to check whether the results from other authors are given as DW (or maybe without drying), and also to have a look at the compounds used as pure standards for the quantification and the methodology applied (extraction protocol and determination conditions). In addition, there are other obvious factors influencing the possible quantitative results, such as the cultivar, the pedoclimatic conditions, the harvesting time, etc. 

In this work, for instance, the adaptability of an olive variety to the pedoclimatic conditions of the site of cultivation could largely condition its leaves metabolites. That could explain the divergence between our results regarding ‘Picual’ and ‘Arbequina’ cv. and those achieved by Talhaoui et al. [26,27]; generally the concentration levels found for some phenolic compounds were higher for the varieties which were cultivated in their country of origin (Spain, in this case). The same is applicable to underline that ‘Picholine Marocaine’ proved to be the cultivar (from the 11 selected herewith) with the highest quantity of phenolic compounds, possibly due to the fact that it is a Moroccan autochthonous variety with verified high adaptability to Moroccan environmental conditions.

When exploring the profile of phenolic compounds present in the studied samples (Table 2, Table 3 and Table 4) to get an idea about their individual (or class) distribution, oleuropein isomer 1 was the prevalent substance in all the analyzed samples regardless of the variety, except for ‘Picual’, in which luteolin-7-glucoside was predominant. Oleuropein, which has been widely investigated for its functional properties as well as its possible recovery and reutilization in various fields [13,32], was the main olive leaf secoiridoid. Oleuropein isomer 1 concentration levels varied from 1632 to 23,963 mg/kg DW, for ‘Picual’ and ‘Picholine Marocaine’ leaves, respectively. Additionally, 2″-methoxyoleuropein isomer 1 was also detected at remarkable levels, fluctuating from 572 (in ‘Picholine Marocaine’) to 2329 mg/kg DW (in ‘Frantoio’). The concentration of some of the other secoiridoids was as follows: Secologanoside isomer 1 (182–1059 mg/kg DW); secologanoside isomer 2 (376–1455 mg/kg DW); elenolic acid glucoside isomer 1 (266–850 mg/kg DW); oleuropein aglycon isomer 1 (48–437 mg/kg DW); elenolic acid glucoside isomer 2 (85–887 mg/kg DW); elenolic acid glucoside isomer 3 (73–989 mg/kg DW); hydroxyoleuropein (147–1027 mg/kg DW) and oleuropein diglucoside (94–623 mg/kg DW). The latter was the minor compound found in samples of 7 varieties (‘Arbequina’, ‘Frantoio’, ‘Lechín’, ‘Manzanilla’, ‘Picholine de Languedoc’, ‘Picual’ and ‘Verdal’), whereas oleuropein aglycon isomer 2 showed the lowest content in leaves from ‘Hojiblanca’, ‘Koroneiki’, ‘Lucque’ and ‘Picholine Marocaine’. It is necessary to emphasize that large standard deviations were obtained for most of the characterized secoiridoids (Table 2, Table 3 and Table 4); that reflects the considerable variability among samples from the same variety. In any case, these intracultivar differences remain rather small when compared with those observed among the studied cultivars. 

A great variability was also observed with regard to flavonoids content. According to Table 2, Table 3 and Table 4, glycosylated flavonoids were much more abundant than aglycone ones. Luteolin-7-glucoside was the major flavonoid compound in the leaves samples of eight varieties (‘Hojiblanca’, ‘Koroneiki’, ‘Lechín’, ‘Lucque’, ‘Manzanilla’, ‘Picholine Marocaine’, ‘Picual’ and ‘Verdal’), with a total concentration range defined by ‘Hojiblanca’ and ‘Lucque’ with values from 2257.5 to 3708.0 mg/kg DW. However, luteolin-glucoside isomer 1 was the predominant glycosylated flavonoid for ‘Arbequina’, ‘Frantoio’ and ‘Picholine de Languedoc’ cultivars; it was found within the overall range 1494–3688 mg/kg DW, defined by ‘Verdal’ and ‘Picholine de Languedoc’ cv. In addition, leaves from ‘Arbequina’ cultivar were characterized by the highest content of luteolin diglucoside (626 mg/kg DW) and chrysoeriol-7-glucoside (606 mg/kg DW), whereas ‘Hojiblanca’ samples exhibited the highest amounts of apigenin rutinoside (542 mg/kg DW) and apigenin-7-glucoside (246 mg/kg DW). Finally, rutin and luteolin-glucoside isomer 2 were prevailing in ‘Lucque’ (2436 mg/kg DW) and ‘Picual’ (364 mg/kg DW) leaves, respectively. In fact, leaves from ‘Lucque’ were outstandingly richest on rutin if compared with samples from the other varieties.

In the sub-category of flavonoids in not-glycosylated form, luteolin was the dominant compound in every case. ‘Arbequina’ leaves showed the highest levels of luteolin (373 mg/kg DW), diosmetin (27 mg/kg DW) and unknown isomer 2 (36 mg/kg DW). ‘Picholine Marocaine’ samples contained the highest amount of quercetin (50 mg/kg DW) and ‘Picholine de Languedoc’ leaves were the richest ones in terms of naringenin (9 mg/kg DW) and unknown isomer 1 (29 mg/kg DW). ‘Koroneiki’ and ‘Hojiblanca’ samples showed the highest content of apigenin (24 mg/kg DW) and unknown isomer 3 (24 mg/kg DW), respectively (Table 2, Table 3 and Table 4). At this point, it is worthy to highlight that this is the first time that the quantification of so many flavonoids derivatives has been performed in olive leaves.

Considering the simple phenols content, the selected varieties could be clustered in two groups: those with hydroxytyrosol as the most abundant simple phenol (‘Arbequina’, ‘Frantoio’, ‘Lucque’, ‘Manzanilla’, ‘Picholine de Languedoc’, ‘Picual’ and ‘Verdal’), and those cultivars with hydroxytyrosol glucoside as the predominant substance within this category (‘Hojiblanca’, ‘Koroneiki’, ‘Lucque’, and ‘Picholine Marocaine’). Hydroxytyrosol levels varied from 119 to 323 mg/kg DW, in ‘Frantoio’ and ‘Picholine Marocaine’, respectively. The latter variety was also the richest regarding hydroxytyrosol glucoside (1510 mg/kg DW), whilst ‘Arbequina’ was the poorest one (10 mg/kg DW). Tyrosol (23–61 mg/kg DW) and tyrosol glucoside (48–237 mg/kg DW) were also found in the samples under study. Vanillic acid and pinoresinol were quantified in the studied olive leaves too. Their concentration levels were relatively low in every sample (<19 mg/kg DW for vanillic acid, and <15 mg/kg DW for pinoresinol) (Table 2, Table 3 and Table 4).

The results of the current study demonstrate that content of individual phenolic compounds in olive leaves is, as expected, closely related to the variety. Indeed, when compared by one-way ANOVA, the contents of the determined compounds were significantly different among the cultivars. Since all the varieties investigated in the current work were grown in the same experimental field using similar agronomic practices, the observed differences regarding the biosynthesis of secondary metabolites can be attributed to the genetic variability. These findings are in good agreement with those reported in literature, as reviewed in detail by Talhaoui and co-workers [24].

Besides, the results of Tukey’s test indicated that individual contents of olive leaves from different cultivars had their own features. Focusing, for instance, on ‘Picholine Marocaine’ traits (Table 4), some specific characteristics can be pointed out. These leaves showed, on average, the highest total phenolic compounds content. This variety is the richest one in terms of secoiridoids (presenting the highest amount of various of these compounds); it presents low concentrations levels of flavonoids in aglycon form, lignans and phenolic acids; however, it contains considerable amounts of simple phenols (in particular, hydroxytyrosol glucoside) and flavonoids in glycosylated form. Thus, it appears that this variety presents, among the other studied cultivars, the greatest potential to be used as plausible source of bioactive compounds, what means that it could be a very promising choice in a future strategy of recycling and valorization of olive leaves from Moroccan olive agro-industry.

### 2.3. Varietal Discrimination 

The genetic diversity of olive trees cultivated all around the world has been explored to identify their varietal origin. Discrimination of the varietal origin of olive trees based on their leaves traits is frequently carried out studying morphological characteristics and genetic markers. Certainly, great advances have been made to explore and prove the usefulness of various olive leaf’s molecular markers, such as amplified fragment length polymorphism, random amplified polymorphic DNA and genomic simple sequence repeat, as reliable tools to differentiate and characterize the genetic diversity of olive cultivars [33,34]. Although these techniques are very valuable, they also have some drawbacks such as complicated pretreatment and DNA extraction procedures, high cost and special requirements for operators. Consequently, there is a need to explore the effectiveness of other analytical approaches to deal with these limitations. The combined application of profiling of olive leaves and chemometrics could be an effective alternative. Hence, in this study, beyond our interest on evaluating the phenolic composition of leaves from different cultivars, we also explored the ability of these compounds to trace the samples varietal origin.

A first attempt to differentiate among the studied varieties was carried out by applying principal components analysis (PCA) to a standardized and centered matrix data, which was constructed with the 38 measured variables (phenolic compounds) and the 55 leaves samples (three extraction replicates). PCA was logically employed as unsupervised method to examine natural grouping of the samples according to their varietal origin in two-dimensional principal components (PCs) plans where each PC is a linear correlation of the original variables (latent variable), and each PC is orthogonal to any other. In this manner, this method studies data structure in a reduced dimension, covering the maximum amount of the information present in the original dataset.

**Figure 3:**
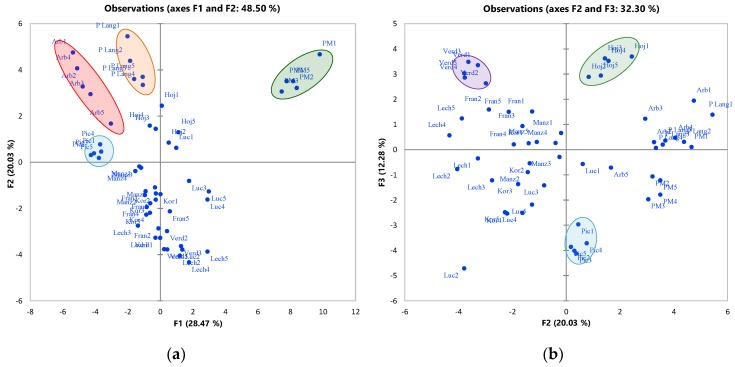
Scatter plot of the PCA scores projected on PC1, PC2 (**a**) and PC2, PC3 (**b**). Abbreviations meaning as in Figure 2. (Even though the statistical treatment was carried out considering the independent extracts and injections of each sample, just the mean value was represented here to facilitate the visual inspection of the figure).

Thus, PCA on leaves phenolic composition resulted in eight PCs with eigenvalues > 1 (PC1 = 10.82; PC2 = 7.61; PC3 = 4.66; PC4 = 3.35; PC5 = 2.47; PC6 = 2.22; PC7 = 1.69 and PC8 = 1.23) that accounted for 89.60% of the total variance of the original result data matrix. Despite the relatively low explained variability retained in the three first PCs (60.77%), the explorative analysis of the projections on the first three PCs (PC1 vs. PC2 (Figure 3a) and PC2 vs. PC3 (Figure 3b)) was crucial to check possible clustering of the leaves samples according to their varietal origin based on their phenolic composition. The results given in Figure 3 show that good separation of 6 varieties could be achieved with a simple PCA (‘Arbequina’, ‘Hojiblanca’, ‘Picholine de Languedoc’, ‘Picholine Marocaine’, ‘Picual’ and ‘Verdal’); the other varieties appeared barely separated in the projections (PC1 vs. PC2 and PC2 vs. PC3).

Subsequently, the potential of applying a supervised multivariate method (stepwise linear discriminant analysis (s-LDA)) was tested. The applicability of the method was cross-validated by using the leave-one-out procedure. The Wilks λ value (0.000) showed that the model was very discriminating, and, in addition, revealed that the probability of correct classification was very high, considering that the *p* value was very low (*p* < 0.0001). Moreover, the forward stepwise statistics, with F-to-enter equal to 1.0 and F-to-remove equal to 0.5, selected 20 variables to be used in the relevant final models: hydroxytyrosol glucoside, 2″-methoxyoleuropein isomer 2, apigenin-7-glucoside, unknown isomer 1, unknown isomer 2, unknown isomer 3, elenolic acid glucoside isomer 1, elenolic acid glucoside isomer 2, ligstroside, ligstroside aglycon, luteolin, luteolin diglucoside, luteolin-glucoside isomer 1, oleuropein aglycon isomer 1, oleuropein isomer 2, oleuropein isomer 3, rutin, secologanoside isomer 1, secologanoside isomer 2 and tyrosol glucoside. 

The results of s-LDA classification and prediction are summarized in the confusion matrices shown in Table 5, displaying re-allocation of samples coming from a given cultivar (corresponding to a matrix row) into the possible categories (the columns). As can be seen from this table, the s-LDA discriminant functions achieved very satisfactory recognition and prediction abilities, being the overall correct rate in both cases 100%. Accordingly, it is possible to assert that the olive leaves phenolic content could be useful for olive cultivars differentiation.

## 3. Materials and Methods 

### 3.1. Olive Leaves Sampling and Preparation

In order to avoid any possible influence of the environmental and agricultural management practices on the obtained results, all olive leaves samples were collected at an experimental orchard in the National School of Agriculture of Meknès in Northern Morocco. Sampling was performed in December 2015, coinciding with the harvesting season in Meknès region, when olive leaves are available as an olive oil processing by-product. This region has a Mediterranean climate type with an average pluviometry of 660 mm/year, and hot and dry summers (maximum temperature up to 40 °C). All necessary agronomic practices (pruning, irrigation, fertilization and pest management) were done according to current olive orchards management standards. Olive trees were vase-trained at a spacing of 7 × 5 m.

Eleven different cultivars were included in this study: a Moroccan autochthonous and predominant variety so-called ‘Picholine Marocaine’, and ten Mediterranean cultivars recently introduced in Morocco (‘Arbequina’, ‘Hojiblanca’, ‘Frantoio’, ‘Koroneiki’, ‘Lechín’, ‘Lucque’, ‘Manzanilla’, ‘Picholine de Languedoc’, ‘Picual’ and ‘Verdal’). Five olive leaves samples per cultivar were randomly collected from cardinally-oriented branches with different directions around the tree’s canopy. Accordingly, a total of 55 olive leaves samples were considered in this work. The leaves were dried at room temperature to constant weight during several days. Once their water content was less than 3%, samples were finely ground in a kind of coffee grinder (but controlling the temperature). Average moisture was calculated after drying different samples in a desiccation oven for 12 h at 100 °C (these tests were just valid to assess the olive leaves moisture; the extraction protocol was obviously not applied to the resulting dried olive leaves). Pre-treated samples were stored in sealed containers and kept below −20 °C in the absence of light till analyzed.

A QC sample was prepared by mixing an equivalent amount of each one of the studied samples; it was used for different purposes: To optimize the extraction procedure, to ensure the proper performance of the analytical system, and to evaluate the analytical parameters of the method. 

### 3.2. Phenolic Compounds Profiling

#### 3.2.1. Chemical and Reagents 

All the chemicals used in this study were of analytical grade. Water was daily deionized by using a Milli-Q system from Millipore (Bedford, MA, USA). Ethanol was supplied by J.T. Baker (Deventer, The Netherlands). Methanol and acetonitrile, both of LC-MS grade, were purchased from Prolabo (Paris, France). Acetic acid and pure standards of apigenin, apigenin-7-glucoside, hydroxytyrosol, luteolin, luteolin-7-glucoside, pinoresinol, rutin, tyrosol and vanillic acid were acquired from Sigma-Aldrich (St. Louis, MO, USA); whereas oleuropein was purchased from Extrasynthese (Lyon, France).

A stock standard solution was prepared by dissolving the appropriate amount of each compound in methanol. Then, diluted working solutions were obtained at nine different concentrations (0.5 mg/L; 1 mg/L; 2.5 mg/L; 5 mg/L; 12.5 mg/L; 25 mg/L; 50 mg/L; 100 mg/L and 200 mg/L) and were stored at −20 °C. If any other concentration level was required for a particular sample or to establish the analytical parameters of the method, it was logically prepared.

#### 3.2.2. Phenolic Compounds Extraction

Pre-treated olive leaves were taken from the freezer and sieved through a 0.5 mm metal sieve, to obtain a standard particle size. 0.1 g of each powdered sample were accurately weighed into a centrifuge tube with a screw cap, and 10 mL of ethanol-water (80:20, *v*/*v*) were added. Then, the mixture was vortexed for 45 s and sonicated for 30 min in an ultrasonic bath from J.P. Selecta (Barcelona, Spain). The resulting extract was centrifuged for 5 min at 5974 g, the supernatant was collected and the residue was re-extracted again following the same procedure as above. Both supernatants were pooled and evaporated to dryness under reduced pressure at 35 °C in a rotavap R-210 (Buchi Labortechnik AG, Flawil, Switzerland). Next, the residue was reconstituted with 5 mL methanol, filtered through a 0.22 μm Nylaflo™ nylon membrane filter from Pall Corporation (Ann Arbor, MI, USA) and subsequently analyzed (or stored in a freezer below −20 °C prior to analysis). Each sample was prepared in triplicate. Every sample was extracted and analyzed by LC-MS on the same day (or within 48–72 h approx.). 

#### 3.2.3. Analytical Procedure and MS Conditions

For chromatographic analysis, an Agilent 1200 Series HPLC system (Agilent Technologies, Santa Clara, CA, USA) operated by Windows NT based ChemStation software and equipped with a binary solvent pump, a degasser, an autosampler, a column oven and a diode array detector (DAD) was used. Separation was performed on a Zorbax C18 analytical column (4.6 × 150 mm, 1.8 μm particle size) from Agilent Technologies (Santa Clara, CA, USA) protected by a guard cartridge and maintained at 25 °C. Injection volume was set at 5 μL. Phenolic compounds elution was achieved with 0.5% acetic acid in water (Phase A) and acetonitrile (Phase B) at a flow rate of 0.8 mL/min and the following gradient program: 0 to 25 min, 5–50% B; 25 to 27 min, 50–95% B; 27 to 27.5 min, 95–100% B; finally, the B content was decreased to the initial conditions (5%) in 1 min and the column was re-equilibrated for 0.5 min prior to the next injection. Double on-line detection was carried out using a DAD (with 240 nm, 254 nm, 280 nm and 330 nm as selected wavelengths) and a mass spectrometer. 

MS analyses were made using two mass spectrometers (both running in negative ionization mode). The first one, a micrOTOF-Q II^TM^ (Bruker Daltonik, Bremen, Germany) equipped with a quadrupole-time-of-flight (Q-TOF) analyzer and an electrospray ionization interface (ESI), was used to investigate the phenolic extracts of the studied olive leaves and to identify as many compounds as possible within the profiles. For this purpose, mixtures of all the extracts coming from the same variety (prepared by mixing an equivalent volume of each one) and the QC sample were analyzed by using this platform. External MS calibration was performed using a 74900-00-05 Cole Palmer syringe pump (manufactory, Vernon Hills, ID, USA) directly connected to the interface, equipped with a Hamilton (Reno, NV, USA) syringe. The calibration solution (sodium formate cluster containing 5 mM sodium hydroxide in the sheath liquid of 0.2% formic acid in water/isopropanol 1:1 *v*/*v*) was injected at the beginning of the run, and all the spectra were calibrated prior to compound identification. The other MS platform was a Bruker Daltonic Esquire 2000™ Ion Trap (IT) mass spectrometer (Bruker Daltonik), which was also coupled to the LC system through an ESI source. This coupling was used to carry out the quantification of the identified substances in all the samples under study. 

For both MS detectors, the flow eluting from the LC column was split using a flow divisor 1:4, so that the flow rate entering into the MS detector was approximately 0.2 mL/min. The following source parameters were adopted for IT MS (and equivalent ones for Q-TOF MS): Capillary voltage, 3200 V; drying gas (N_2_) flow and temperature, 9 L/min and 300 °C, respectively; nebulizer pressure, 30 psi. In IT MS, Ion Charge Control (ICC) was set at 10,000 and 50–1000 *m*/*z* was the selected scan range. Instrument control and data processing were carried out using the software Esquire Control and Data Analysis 4.0, respectively (Bruker Daltonik).

Quantitative determinations were carried out using the calibration curves obtained from commercially available pure standards. The results were expressed as mg of analyte/kg of olive leaves dry weight (DW). 

### 3.3. Statistical Analysis

All data were reported as mean ± standard deviation (*n* = 5, corresponding to the number of samples per studied cultivar). Comparisons between means were performed by applying One-way Analysis of Variance (ANOVA) with Tukey’s *post-hoc* test, using IBM SPSS Statistics 20 (SPSS Inc., Chicago, IL, USA). The differences between studied varieties were considered significant with *p* < 0.05. Furthermore, PCA and s-LDA were performed on phenolic compounds quantitative data to assess the potential of these substances to discriminate the studied samples according to their varietal origin. Multivariate data analysis was performed with the Microsoft Office Excel 2016 software (Microsoft Corporation, Redmon, WA, USA) and the statistical software XLSTAT version 2015.04.1 (Addinsoft, Paris, France).

## 4. Conclusions

The achieved results demonstrated—in the Moroccan context—the potential of the olive leaves as an underexploited natural source of interesting substances with inherent applications in different fields; their recovery could be a valuable alternative for the sustainable and environmentally friendly management of olive leaves mills by-products. 

In Morocco, olive orchards are predominantly planted with ‘Picholine Marocaine’ variety. In 2015 about 1.15 million tons of olive fruits were harvested; olive leaves represented on average 6% of harvested olive fruits, which means about 27.6–34.5 thousand tons of dry olive leaves. Considering our results (for the autochthonous Moroccan cv. in particular), they could potentially contain around 650–825 tons of oleuropein, which are actually wasted. It is time to establish an integrated approach for the sustainable extraction of high value-added molecules from olive leaves in Morocco.

Apart from the clear future practical application of this work (isolation of the bioactive compounds of interest such as oleuropein), it is important to highlight that the comprehensive methodology used, combining LC-MS data on phenolic compounds and related substances with chemometrics, resulted to be a very effective tool for achieving an adequate discrimination among the olive leaves from different cultivars.

## Figures and Tables

**Table 1:** Main phenolic compounds tentatively identified in the olive leaves from the 11 different selected varieties using the optimized LC-ESI-Q-TOF MS profiling approach.

Peak	Retention Time	Molecular Formula	Experimental *m*/z *	Calculated *m*/*z*	Error (ppm)	mSigma	Suggested Compound
**1**	6.4	C_14_H_20_O_8_	315.1083	315.1085	0.8	6.2	Hydroxytyrosol glucoside
**2**	6.7	C_16_H_22_O_11_	389.1086	389.1089	1.0	5.2	Secologanoside is. 1
**3**	7.3	C_8_H_10_O_3_	153.0557	153.0557	0.1	8.7	Hydroxytyrosol
**4**	8.1	C_14_H_20_O_7_	299.1131	299.1136	1.8	2.3	Tyrosol glucoside
**5**	9.2	C_16_H_22_O_11_	389.1088	389.1089	0.3	18	Secologanoside is. 2
**6**	9.4	C_8_H_10_O_2_	137.0607	137.0608	1.0	8.2	Tyrosol
**7**	10.6	C_17_H_24_O_11_	403.1247	403.1246	−0.2	6.3	Elenolic acid glucoside is. 1
**8**	10.8	C_8_H_8_O_4_	167.0348	167.035	1.1	3.1	Vanillic acid
**9**	10.9	C_19_H_22_O_8_	377.1446	377.1453	2.0	6.9	Oleuropein aglycon is. 1
**10**	11.1	C_27_H_30_O_16_	609.1468	609.1461	−1.2	21.4	Luteolin diglucoside
**11**	11.9	C_17_H_24_O_11_	403.1246	403.1246	0	15.5	Elenolic acid glucoside is. 2
**12**	12.5	C_17_H_24_O_11_	403.1239	403.1246	1.8	10.2	Elenolic acid glucoside is. 3
**13**	13.2	C_27_H_30_O_16_	609.146	609.1461	0.1	3.1	Rutin
**14**	13.3	C_25_H_32_O_14_	555.1707	555.1719	2.2	6.2	Hydroxyoleuropein
**15**	13.9	C_21_H_20_O_11_	447.0934	447.0933	−0.3	11	Luteolin-7-glucoside
**16**	14.5	C_27_H_30_O_14_	577.157	577.1563	−1.3	19.3	Apigenin rutinoside
**17**	14.7	C_31_H_42_O_18_	701.2299	701.2298	0	5.5	Oleuropein diglucoside
**18**	15. 5	C_21_H_20_O_10_	431.0983	431.0984	0.2	4.9	Apigenin-7-glucoside
**19**	15.6	C_21_H_20_O_11_	447.0938	447.0933	−1.1	8.4	Luteolin-glucoside is. 1
**20**	15.7	C_22_H_22_O_11_	461.1086	461.1089	0.7	13.9	Chrysoeriol-7-glucoside
**21**	16.3	C_21_H_20_O_11_	447.0941	447.0933	−1.8	8.1	Luteolin-glucoside is. 2
**22**	16.3	C_26_H_34_O_14_	569.1869	569.1876	1.3	24.2	2″-methoxyoleuropein is. 1
**23**	16.7	C_25_H_32_O_13_	539.1769	539.1770	0.2	12.6	Oleuropein is. 1
**24**	17.0	C_26_H_34_O_14_	569.1875	569.1876	0.1	2.6	2″-methoxyoleuropein is. 2
**25**	17.1	C_25_H_32_O_13_	539.1766	539.1771	0.9	8.3	Oleuropein is. 2
**26**	17.4	C_25_H_32_O_13_	539.1765	539.1769	0.7	4.7	Oleuropein is. 3
**27**	18.5	C_25_H_32_O_12_	523.1812	523.1821	1.8	21.5	Ligstroside
**28**	19.3	C_19_H_22_O_7_	361.1287	361.1293	1.5	2.7	Ligtroside aglycone
**29**	19.8	C_15_H_10_O_6_	285.0399	285.0405	2.0	16.3	Luteolin
**30**	20.1	C_15_H_10_O_7_	301.0354	301.0354	0	7.7	Quercetin
**31**	20.5	C_20_H_22_O_6_	357.1355	357.1344	−3.2	3	Pinoresinol
**32**	22.3	C_15_H_10_O_5_	269.0456	269.0455	−0.3	7.4	Apigenin
**33**	22.5	C_15_H_12_O_5_	271.0612	271.0612	−0.1	13.6	Naringenin
**34**	22.8	C_16_H_12_O_6_	299.0564	299.0561	−1.0	16.2	Diosmetin
**35**	23.3	C_15_H_8_O_7_	299.0202	299.0197	−1.4	12.3	Uk is. 1
**36**	24.1	C_19_ H_22_O_8_	377.1242	377.1242	−0.1	17.1	Oleuropein aglycon is. 2
**37**	26.0	C_15_H_8_O_7_	299.0196	299.0197	0.4	6.1	Uk is. 2
**38**	26.7	C_15_H_8_O_7_	299.0200	299.0197	−0.9	13.9	Uk is. 3

* *m*/*z* values correspond to [M − H]^−^ in every case. is.: Isomer; Uk: Unknown.

**Table 2:** Found content (average values and standard deviation, mg/kg DW) of the determined phenolic compounds in the evaluated olive leaves cultivars. ANOVA results are included; significant differences in the same row are indicated with different superscript letters (comparison among the 11 cultivars investigated in this study, *p* < 0.05).

	‘Arbequina’	‘Frantoio’	‘Hojiblanca’	‘Koroneiki’
Hydroxytyrosol glucoside	10 ^a^ ± 5	22 ^a^ ± 7	185 ^b^ ± 33	203 ^b^ ± 16
Secologanoside is. 1	333 ^ab^ ± 28	754 ^e^ ± 120	844 ^ef^ ± 80	643 ^de^ ± 42
Hydroxytyrosol	209 ^a^ ± 39	119 ^b^ ± 14	147 ^ab^ ± 23	136 ^b^ ± 7
Tyrosol glucoside	61 ^f^ ± 5	48 ^ef^ ± 6	120 ^d^ ± 13	178 ^b^ ± 20
Secologanoside is. 2	483 ^ac^ ± 67	1312 ^bd^ ± 80	1330 ^bd^ ± 69	769 ^ce^ ± 184
Tyrosol	53 ^ab^ ± 10	29 ^cd^ ± 5	31 ^cd^ ± 3	41 ^bd^ ± 7
Elenolic acid glucoside is. 1	484 ^d^ ± 43	850 ^c^ ± 63	742 ^b^ ± 34	576 ^d^ ± 38
Vanillic acid	19 ^a^ ± 3	9 ^c^ ± 2	13 ^bc^ ± 2	12 ^c^ ± 2
Oleuropein aglycon is. 1	48 ^a^ ± 11	422 ^b^ ± 60	206 ^cd^ ± 20	397 ^b^ ± 38
Luteolin diglucoside	626 ^a^ ± 79	421 ^c^ ± 66	240 ^b^ ± 26	355 ^bc^ ± 36
Elenolic acid glucoside is. 2	95 ^b^ ± 13	468 ^ef^ ± 104	431 ^def^ ± 15	370 ^def^ ± 27
Elenolic acid glucoside is. 3	73 ^c^ ± 4	174 ^e^ ± 14	140 ^de^ ± 17	135 ^de^ ± 20
Rutin	411 ^de^ ± 34	542 ^ce^ ± 113	489 ^ce^ ± 53	1099 ^a^ ± 223
Hydroxyoleuropein	525 ^cf^ ± 54	758 ^de^ ± 90	757 ^de^ ± 48	843 ^d^ ± 43
Luteolin-7-glucoside	3324 ^ab^ ± 375	2527 ^c^ ± 408	3708 ^a^ ± 322	2632 ^c^ ± 191
Apigenin rutinoside	431 ^def^ ± 60	354 ^bdf^ ± 36	542 ^a^ ± 65	312 ^bcf^ ± 46
Oleuropein diglucoside	94 ^c^ ± 19	249 ^efh^ ± 36	458 ^a^ ± 24	301 ^df^ ± 38
Apigenin-7-glucoside	65 ^bc^ ± 12	65 ^bc^ ± 6	246 ^ad^ ± 13	158 ^c^ ± 23
Luteolin-glucoside is. 1	3428 ^ac^ ± 542	3013 ^abc^ ± 555	3584 ^c^ ± 172	1630 ^de^ ± 513
Chrysoeriol-7-glucoside	606 ^b^ ± 44	496 ^cd^ ± 27	552 ^bc^ ± 24	387 ^a^ ± 41
Luteolin-glucoside is. 2	295 ^cdfgh^ ± 32	341 ^dgh^ ± 49	230 ^cbf^ ± 12	347 ^degh^ ± 50
2″-methoxyoleuropein is.1	1499 ^bd^ ± 194	2329 ^a^ ± 231	2063 ^ad^ ± 159	1642 ^bd^ ± 510
Oleuropein is. 1	3465 ^e^ ± 960	10,959 ^cdf^ ± 3283	6923 ^def^ ± 1813	6023 ^def^ ± 1679
2″-methoxyoleuropein is. 2	130 ^e^ ± 31	100 ^de^ ± 16	176 ^a^ ± 22	128 ^e^ ± 31
Oleuropein is. 2	57 ^ce^±21	159 ^def^ ± 51	130 ^cf^ ± 54	139 ^cf^ ± 60
Oleuropein is. 3	234 ^cf^ ± 50	336 ^cf^ ± 106	440 ^df^ ± 116	375 ^ef^ ± 96
Ligstroside	505 ^df^ ± 92	343 ^cd^ ± 51	406 ^cd^ ± 37	496 ^de^ ± 130
Ligstroside aglycon	334 ^bc^ ± 93	142 ^c^ ± 104	312 ^c^ ± 41	278 ^c^ ± 33
Luteolin	373 ^a^ ± 63	189 ^e^ ± 23	175 ^de^ ± 20	279 ^b^ ± 34
Quercetin	41 ^a^ ± 11	14 ^b^ ± 3	14 ^b^ ± 1	9 ^b^ ± 5
Pinoresinol	11 ^bcde^ ± 1	16 ^a^ ± 3	12 ^bce^ ± 1	11.1 ^bcde^ ± 0.7
Apigenin	21 ^bc^ ± 6	12 ^acdf^ ± 2	17 ^bde^ ± 2	24 ^b^ ± 11
Naringenin	7 ^ac^ ± 1	5.4 ^c^ ± 0.7	6 ^bc^ ± 1	5.3 ^c^ ± 0.4
Diosmetin	27 ^a^ ± 7	14 ^cd^ ± 2	6.2 ^b^ ± 0.7	15 ^cd^ ± 2
Unknown is. 1	13 ^efg^ ± 3	13 ^ef^ ± 2	21 ^d^ ± 2	3 ^b^ ± 3
Oleuropein aglycon is. 2	60 ^e^ ± 36	359 ^bc^ ± 66	18 ^e^ ± 5	13 ^e^ ± 5
Unknown is. 2	36 ^a^ ± 6	14 ^cd^ ± 2	28 ^a^ ± 2	8 ^bc^ ± 4
Unknown is. 3	14 ^a^ ± 4	12 ^a^ ± 2	24 ^a^ ± 3	6 ^bc^ ± 1

**Table 3:** Found content (average values and standard deviation, mg/kg DW) of the determined phenolic compounds in the evaluated olive leaves cultivars. ANOVA results are included; significant differences in the same row are indicated with different superscript letters (comparison among the 11 cultivars investigated in this study, *p* < 0.05).

	‘Lechin’	‘Lucque’	‘Manzanilla’	‘Picholine de Languedoc’
Hydroxytyrosol glucoside	39 ^a^ ± 19	316 ^c^ ± 64	48 ^a^ ± 7	186 ^b^ ± 18
Secologanoside is. 1	876 ^cef^ ± 198	1018 ^cf^ ± 91	507 ^bd^ ± 74	608 ^de^ ± 49
Hydroxytyrosol	147 ^ab^ ± 42	143 ^ab^ ± 58	144 ^ab^ ± 20	202 ^a^ ± 46
Tyrosol glucoside	122 ^cd^ ± 28	114 ^cd^ ± 22	60 ^efg^ ± 2	160 ^b^ ± 19
Secologanoside is. 2	1455 ^b^ ± 298	854 ^e^ ± 132	746 ^ce^ ± 125	572 ^ace^ ± 61
Tyrosol	38 ^d^ ± 4	23 ^c^ ± 6	44 ^bd^ ± 4	33 ^cd^ ± 4
Elenolic acid glucoside is. 1	799 ^bc^ ± 99	507 ^d^ ± 48	513 ^d^ ± 15	494 ^d^ ± 24
Vanillic acid	10 ^c^ ± 3	9 ^c^ ± 2	10 ^c^ ± 2	18 ^ab^ ± 5
Oleuropein aglycon is. 1	143 ^de^ ± 37	244 ^c^ ± 57	202 ^cd^ ± 12	164 ^de^ ± 25
Luteolin diglucoside	393 ^c^ ± 48	302 ^bc^ ± 104	344 ^bc^ ± 31	607 ^a^ ± 102
Elenolic acid glucoside is. 2	323 ^df^ ± 95	346 ^cdef^ ± 49	226 ^cd^ ± 29	426 ^f^ ± 27
Elenolic acid glucoside is. 3	93 ^cd^ ± 12	153 ^de^ ± 22	156 ^e^ ± 18	264 ^a^ ± 33
Rutin	294 ^de^ ± 26	2436 ^b^ ± 320	384 ^de^ ± 67	689 ^c^ ± 82
Hydroxyoleuropein	577 ^fg^ ± 41	551 ^cf^ ± 83	643 ^ef^ ± 17	490 ^cg^ ± 33
Luteolin-7-glucoside	2715 ^bc^ ± 101	2258 ^c^ ± 561	2561 ^c^ ± 223	3548 ^a^ ± 358
Apigenin rutinoside	275 ^b^ ± 15	385 ^cdef^ ± 56	471 ^aef^ ± 32	396 ^cdef^ ± 39
Oleuropein diglucoside	164 ^cg^ ± 38	312 ^dfh^ ± 60	219 ^eg^ ± 27	354 ^d^ ± 20
Apigenin-7-glucoside	93 ^f^ ± 3	221 ^ae^ ± 42	157 ^d^ ± 5	135 ^df^ ± 6
Luteolin-glucoside is. 1	2289 ^bde^ ± 250	2598 ^ab^ ± 965	2425 ^bde^ ± 271	3687 ^c^ ± 266
Chrysoeriol-7-glucoside	532 ^bc^ ± 30	498 ^cd^ ± 54	437 ^ad^ ± 83	547 ^bc^ ± 23
Luteolin-glucoside is. 2	350 ^degh^ ± 43	267 ^fg^ ± 21	209 ^bf^ ± 24	317 ^gh^ ± 40
2″-methoxyoleuropein is.1	1588 ^bd^ ± 255	761 ^ce^ ± 314	1162 ^bc^ ± 213	928 ^ce^ ± 86
Oleuropein is. 1	20,645 ^ab^ ± 8348	1535 ^bc^ ± 2708	7696 ^def^ ± 1583	8176 ^def^ ± 895
2″-methoxyoleuropein is. 2	67 ^bd^ ± 8	54 ^bc^ ± 14	100 ^de^ ± 21	133 ^e^ ± 10
Oleuropein is. 2	247 ^bd^ ± 85	301 ^b^ ± 54	115 ^cf^ ± 42	174 ^df^ ± 22
Oleuropein is. 3	638 ^bd^ ± 197	873 ^b^ ± 207	397 ^df^ ± 107	597 ^de^ ± 67
Ligstroside	653 ^d^ ± 147	425 ^cd^ ± 28	575 ^d^ ± 31	185 ^cef^ ± 22
Ligstroside aglycon	979 ^a^ ± 494	400 ^bc^ ± 113	526 ^bc^ ± 185	447 ^bc^ ± 104
Luteolin	169 ^de^ ± 35	113 ^cd^ ± 57	157 ^de^ ± 18	276 ^b^ ± 32
Quercetin	3.9 ^b^ ± 0.6	7 ^b^ ± 4	10 ^b^ ± 2	19 ^b^ ± 3
Pinoresinol	9.6 ^cde^ ± 0.8	8.7 ^d^ ± 0.7	12.8 ^ae^ ± 0.9	11 ^cde^ ± 2
Apigenin	11 ^aef^ ± 2	11 ^aef^ ± 2	16 ^ab^ ± 2	17 ^bf^ ± 3
Naringenin	7 ^ac^ ± 1	5.0 ^c^ ± 0.5	8 ^ab^ ± 2	8.7 ^a^ ± 0.8
Diosmetin	6 ^b^ ± 2	6 ^b^ ± 4	6 ^b^ ± 2	20 ^d^ ± 4
Unknown is. 1	4 ^bc^ ± 2	9 ^ce^ ± 3	16 ^df^ ± 2	29 ^a^ ± 2
Oleuropein aglycon is. 2	666 ^a^ ± 260	53 ^e^ ± 17	262 ^cd^ ± 100	132 ^de^ ± 19
Unknown is. 2	4 ^b^ ± 1	18 ^d^ ± 6	18 ^d^ ± 2	30 ^a^ ± 3
Unknown is. 3	3 ^b^ ± 2	11 ^cd^ ± 3	14 ^d^ ± 2	22.6 ^a^ ± 0.7

**Table 4:** Found content (average values and standard deviation, mg/kg DW) of the determined phenolic compounds in the evaluated olive leaves cultivars. ANOVA results are included; significant differences in the same row are indicated with different superscript letters (comparison among the 11 cultivars investigated in this study, *p* < 0.05).

	‘Picholine Marocaine’	‘Picual’	‘Verdal’
Hydroxytyrosol glucoside	1510 ^d^ ± 67	11 ^a^ ± 6	15 ^a^ ± 9
Secologanoside is. 1	1059 ^c^ ± 50	182 ^a^ ± 38	1005 ^cf^ ± 112
Hydroxytyrosol	323 ^c^ ± 22	155 ^ab^ ± 14	140 ^b^ ± 11
Tyrosol glucoside	237 ^a^ ± 13	62 ^efg^ ± 10	82 ^cefg^ ± 6
Secologanoside is. 2	1199 ^bd^ ± 226	376 ^a^ ± 94	1100 ^de^ ± 144
Tyrosol	54 ^ab^ ± 10	28 ^cd^ ± 4	61 ^a^ ± 8
Elenolic acid glucoside is. 1	342 ^a^ ± 29	266 ^a^ ± 42	787 ^bc^ ± 58
Vanillic acid	7 ^c^ ± 1	19 ^a^ ± 4	8 ^c^ ± 2
Oleuropein aglycon is. 1	437 ^b^ ± 37	105 ^ae^ ± 41	173 ^cde^ ± 23
Luteolin diglucoside	395 ^c^ ± 31	353 ^bc^ ± 38	294 ^bc^ ± 27
Elenolic acid glucoside is. 2	887 ^a^ ± 95	85 ^b^ ± 19	402 ^cdef^ ± 65
Elenolic acid glucoside is. 3	989 ^b^ ± 75	114 ^ce^ ± 11	127 ^ce^ ± 17
Rutin	554 ^ce^ ± 45	161 ^d^ ± 28	362 ^de^ ± 17
Hydroxyoleuropein	147 ^a^ ± 16	420 ^c^ ± 70	1027 ^b^ ± 140
Luteolin-7-glucoside	2800 ^bc^ ± 232	2284 ^c^ ± 152	2662 ^bc^ ± 292
Apigenin rutinoside	456 ^ade^ ± 32	395 ^cdef^ ± 80	327 ^f^ ± 45
Oleuropein diglucoside	623 ^b^ ± 47	94 ^c^ ± 38	243 ^efg^ ± 28
Apigenin-7-glucoside	148 ^df^ ± 18	114 ^f^ ± 7	202 ^e^ ± 11
Luteolin-glucoside is. 1	2471 ^bd^ ± 228	2132 ^de^ ± 130	1494 ^e^ ± 115
Chrysoeriol-7-glucoside	480 ^cd^ ± 26	424 ^ad^ ± 14	495 ^ad^ ± 12
Luteolin-glucoside is. 2	277 ^befgh^ ± 36	364 ^h^ ± 58	116 ^a^ ± 7
2″-methoxyoleuropein is.1	572 ^e^ ± 48	611 ^ce^ ± 188	2241 ^a^ ± 384
Oleuropein is. 1	23,963 ^a^ ± 3513	1632 ^e^ ± 437	12,443 ^cf^ ± 2403
2″-methoxyoleuropein is. 2	127 ^e^ ± 10	52 ^b^ ± 19	95 ^cde^ ± 16
Oleuropein is. 2	434 ^a^ ± 47	42 ^c^ ± 15	193 ^df^ ± 54
Oleuropein is. 3	2249 ^a^ ± 126	114 ^c^ ± 40	419 ^df^ ± 87
Ligstroside	1118 ^a^ ± 358	129 ^c^ ± 55	1608 ^b^ ± 260
Ligstroside aglycon	209 ^c^ ± 20	298 ^c^ ± 39	730 ^ab^ ± 300
Luteolin	49 ^c^ ± 8	265 ^b^ ± 28	184 ^de^ ± 16
Quercetin	50 ± 14	7 ^b^ ± 2	7 ^b^ ± 2
Pinoresinol	10.1 ^bcde^ ± 0.6	10 ^cde^ ± 1	14 ^ab^ ± 1
Apigenin	7.5 ^ac^ ± 0.7	21 ^b^ ± 2	19 ^bf^ ± 3
Naringenin	5.2 ^c^ ± 0.5	6.6 ^ac^ ± 0.5	6.3 ^ac^ ± 0.3
Diosmetin	4 ^b^ ± 1	16 ^cd^ ± 2	13 ^c^ ± 2
Unknown is. 1	19 ^dg^ ± 4	16 ^df^ ± 2	10 ^e^ ± 2
Oleuropein aglycon is. 2	125 ^de^ ± 18	32 ^e^ ± 12	465 ^b^ ± 91
Unknown is. 2	15 ^cd^ ± 3	18 ^d^ ± 3	16 ^d^ ± 2
Unknown is. 3	15 ^d^ ± 2	13 ^d^ ± 1	12 ^cd^ ± 2

**Table 5:** Classification and Prediction ability results of s-LDA model, based on olive leaves phenolic composition, for achieving varietal origin separation.

**Confusion Matrix for the Training Sample**													
**Variety/Classified as**	**Arbequina**	**Frantoio**	**Hojiblanca**	**Koroneiki**	**Lechín**	**Lucque**	**Manzanilla**	**Picholine Marocaine**	**Picholine de Languedoc**	**Picual**	**Verdal**	**Total**	**% Correct**
Arbequina	5	0	0	0	0	0	0	0	0	0	0	5	100.0
Frantoio	0	5	0	0	0	0	0	0	0	0	0	5	100.0
Hojiblanca	0	0	5	0	0	0	0	0	0	0	0	5	100.0
Koroneiki	0	0	0	5	0	0	0	0	0	0	0	5	100.0
Lechín	0	0	0	0	5	0	0	0	0	0	0	5	100.0
Lucque	0	0	0	0	0	5	0	0	0	0	0	5	100.0
Manzanilla	0	0	0	0	0	0	5	0	0	0	0	5	100.0
Picholine Marocaine	0	0	0	0	0	0	0	5	0	0	0	5	100.0
Picholine de Languedoc	0	0	0	0	0	0	0	0	5	0	0	5	100.0
Picual	0	0	0	0	0	0	0	0	0	5	0	5	100.0
Verdal	0	0	0	0	0	0	0	0	0	0	5	5	100.0
Total	5	5	5	5	5	5	5	5	5	5	5	55	100.0
**Confusion Matrix for the Cross-Validation Results**													
**Variety/Classified as**	**Arbequina**	**Frantoio**	**Hojiblanca**	**Koroneiki**	**Lechín**	**Lucque**	**Manzanilla**	**Picholine Marocaine**	**Picholine de Languedoc**	**Picual**	**Verdal**	**Total**	**% Correct**
Arbequina	5	0	0	0	0	0	0	0	0	0	0	5	100.0
Frantoio	0	5	0	0	0	0	0	0	0	0	0	5	100.0
Hojiblanca	0	0	5	0	0	0	0	0	0	0	0	5	100.0
Koroneiki	0	0	0	5	0	0	0	0	0	0	0	5	100.0
Lechín	0	0	0	0	5	0	0	0	0	0	0	5	100.0
Lucque	0	0	0	0	0	5	0	0	0	0	0	5	100.0
Manzanilla	0	0	0	0	0	0	5	0	0	0	0	5	100.0
Picholine Marocaine	0	0	0	0	0	0	0	5	0	0	0	5	100.0
Picholine de Languedoc	0	0	0	0	0	0	0	0	5	0	0	5	100.0
Picual	0	0	0	0	0	0	0	0	0	5	0	5	100.0
Verdal	0	0	0	0	0	0	0	0	0	0	5	5	100.0
Total	5	5	5	5	5	5	5	5	5	5	5	55	100.0

## Supplementary Materials

The following are available online, Table S1: Analytical parameters of the developed LC-MS method, including calibration curves equations and r^2^, LOD and LOQ, linear ranges and repeatability (expressed as %RSD).
