# Supplementary material for: Establishing the Phenolic Composition of Olea europaea L. Leaves from Cultivars Grown in Morocco as a Crucial Step Towards Their Subsequent Exploitation
Source: Molecules. 2018 Oct 2;23(10):2524. doi: 10.3390/molecules23102524 (PMC6222472; doi:10.3390/molecules23102524)
Supplement: Supplementary file 1 [file molecules-23-02524-s001.pdf]

Supplementary Materials

Table 1S. Analytical parameters of the developed LC-MS method, including calibration curves equations and r^2, LOD and LOQ, linear ranges and repeatability (expressed as % RSD).

Compound	Rt (min)	Calibration curves	r^2	LOD (µg/L)	LOQ (µg/L)	Linear range[a] (mg/L)	Repeatability[b] RSD*intra-day* (%)	RSD*inter-day* (%)
**Hydroxytyrosol**	7.3	y = 29083x + 7653.4	0.9985	14	45	60	2.1	5.6
**Tyrosol**	9.4	y = 11972x - 1367.2	0.9999	42	141	70	5.1	7.1
**Vanillic acid**	10.8	y = 13497x - 636.8	0.9984	36	120	5	4.3	5.5
**Rutin**	13.2	y = 3420x - 902.06	0.9957	97	325	12.5	4.4	9.4
**Luteolin-7-glucoside**	13.9	y = 9917x + 6620.8	0.9951	41	136	12.5	7.5	8.6
**Apigenin-7-glucoside**	15.5	y = 19166x + 15207	0.9918	15	51	12.5	5.8	6.7
**Oleuropein**	16.7	y = 8630.3x + 509.45	0.9981	40	132	12.5	3.8	3.9
**Luteolin**	19.8	y = 127143x + 7126.3	0.9984	5	18	12.5	3.4	3.8
**Pinoresinol**	20.5	y = 48060x - 5210.3	0.9996	16	53	12.5	5.1	5.7
**Apigenin**	22.3	y = 190558x - 6919.3	0.9971	3	11	12.5	1.8	2.1

[a]Linear ranges were established from LOQ to the indicated value.
[b]Repeatability is expressed as % RSD of peak area for 4 injections of 4 different extracts of the QC carried out within the same sequence (*intra*-day) or over 4 days (*inter*-day).
